# Data Interoperability in Context: The Importance of Open-Source Implementations When Choosing Open Standards

**DOI:** 10.2196/66616

**Published:** 2025-04-15

**Authors:** Daniel Kapitan, Femke Heddema, André Dekker, Melle Sieswerda, Bart-Jan Verhoeff, Matt Berg

**Affiliations:** 1 Eindhoven AI Systems Institute (EAISI) Eindhoven University of Technology Eindhoven The Netherlands; 2 PharmAccess Foundation Amsterdam The Netherlands; 3 Dutch Hospital Data Utrecht The Netherlands; 4 MAASTRO Clinic Maastricht University Medical Centre Maastricht University Maastricht The Netherlands; 5 Netherlands Comprehensive Cancer Organisation Utrecht The Netherlands; 6 Expertisecentrum Zorgalgoritmen Utrecht The Netherlands; 7 Ona Burlington, VT United States

**Keywords:** FHIR, OMOP, openEHR, health care informatics, information standards, secondary use, digital platform, data sharing, data interoperability, open source implementations, open standards, Fast Health Interoperability Resources, Observational Medical Outcomes Partnership, clinical care, data exchange, longitudinal analysis, low income, middle-income, LMIC, low and middle-income countries, developing countries, developing nations, health information exchange

## Abstract

Following the proposal by Tsafnat et al (2024) to converge on three open health data standards, this viewpoint offers a critical reflection on their proposed alignment of openEHR, Fast Health Interoperability Resources (FHIR), and Observational Medical Outcomes Partnership (OMOP) as default data standards for clinical care and administration, data exchange, and longitudinal analysis, respectively. We argue that open standards are a necessary but not sufficient condition to achieve health data interoperability. The ecosystem of open-source software needs to be considered when choosing an appropriate standard for a given context. We discuss two specific contexts, namely standardization of (1) health data for federated learning, and (2) health data sharing in low- and middle-income countries. Specific design principles, practical considerations, and implementation choices for these two contexts are described, based on ongoing work in both areas. In the case of federated learning, we observe convergence toward OMOP and FHIR, where the two standards can effectively be used side-by-side given the availability of mediators between the two. In the case of health information exchanges in low and middle-income countries, we see a strong convergence toward FHIR as the primary standard. We propose practical guidelines for context-specific adaptation of open health data standards.

## Open Standards Are a Necessary but Not Sufficient Condition for Interoperability

“A paradox of health care interoperability is the existence of a large number of standards with significant overlap among them,” says Tsafnat et al [1] followed by a call to action toward the health informatics community to put effort into establishing convergence and preventing collision. To do so, they propose to converge on three open standards, namely (1) openEHR for clinical care and administration, (2) Fast Health Interoperability Resources (FHIR) for data exchange, and (3) Observational Medical Outcomes Partnership Common Data Model (OMOP) for longitudinal analysis. They argue that open data standards, backed by engaged communities, hold an advantage over proprietary ones, and therefore, should be chosen as the stepping stones toward achieving true interoperability.

While we support their high-level rationale and intention, we feel their proposed trichotomy does not do justice to details that are crucial in real-world implementations. This viewpoint provides a critical reflection on their proposed framework in three parts. First, we reflect on salient differences between the three open standards from the perspective of the notion of openness of digital platforms [2], the paradox of open [3], and the hourglass model of open architectures [4,5]. Subsequently, we outline the importance of open-source software (OSS) by reflecting on our considerations in designing and implementing health data platforms in two specific contexts, namely (1) platforms for federated learning (FL) on shared health data in high-income countries and (2) health data platforms for low- and middle-income countries (LMICs). These case studies illustrate the limitations of the trichotomy proposed by Tsafnat et al [1]. Particularly, we argue that of the 3 standards, FHIR stands out as being the most practical and adaptable which allows it to be used for longitudinal analysis and routine collection of clinical data, besides its original purpose as a health data exchange standard. We conclude this viewpoint with practical implications of these findings and directions for future research of open health data standards.

## Digital Platforms Require Extensibility, Availability of Complementary Components, and Availability of Executable Pieces of Software

In their editorial, Tsafnat et al [1] argue that (1) the paradox of interoperability of having overlapping standards can be addressed by converging on just three standards; (2) practical and sociotechnical considerations are as important as, if not more important than, technical superiority and therefore balancing of customizability and rigidity is of the essence; and (3) open standards, backed by engaged communities, hold an advantage over proprietary ones. While we concur with these points, we argue that these are necessary, but not sufficient conditions for convergence of health data standards. Existing research on digital platforms underlines the importance of the platform’s openness, not only in terms of open standards but also in terms of the availability of executable pieces of software, extensibility of the code base, and availability of complements to the core technical platform (in this case, the health data standard is a critical, defining component of the core technical platform) [2]. Openness in this context pertains to the software modules that constitute the digital platform (Textbox 1). Realizing openness can be achieved through open-sourcing the core components of the platform or defining standardized interfaces through which components can interact [6]. Only when the majority of these aspects of digital platforms are met can we reasonably expect that the digital platform will indeed flourish and be long-lived.

If open digital platforms are what we want, the question is how to achieve that. In what they frame as “the paradox of open,” Keller and Tarkowski [3] argue that open platforms and their associated ecosystems can only flourish if two types of conditions are met. The first condition states that many people need to contribute to the creation of a common resource. “This is the story of Wikipedia, OpenStreetMap, Blender.org, and the countless free software projects that provide much of the internet’s infrastructure” [3]. Indeed, Tsafnat et al [1] have explicitly taken into account that “an engaged and vibrant community is a major advantage for the longevity of the data standards it uses,” which has informed their proposal to converge toward OMOP, FHIR, and openEHR over other existing health data standards. However, the importance of OSS is somewhat overlooked. This point is only mentioned in passing when Tsafnat et al [1] reference work done by Reynolds and Wyatt [7] who already argued in 2011 “… for the superiority of open-source licensing to promote safer, more effective health care information systems. We claim that open-source licensing in health care information systems is essential to rational procurement strategy.” Hence, we extend the line of reasoning of Tsafnat et al [1] by emphasizing that the availability of executable OSS components, which inherently makes it easier to extend the code base of the health data standard and thereby drive greater availability of complementary components, is an important criterion which needs to be explicitly taken into account when choosing which standard to adopt.

The second condition put forward by Keller and Tarkowski [3] is that open ecosystems have proven fruitful when “opening up” is the result of external incentives or requirements, rather than voluntary actions. Examples of such external incentives are “...publicly funded knowledge production like Open Access academic publications, cultural heritage collections in the Public Domain, Open Educational Resources, and Open Government data.” Another canonical example is the birth of the Global System for Mobile Communications standard, which was mandated by European legislation [8]. Reflecting on this condition in the context of open health data ecosystems, we observe a salient difference between FHIR versus openEHR and OMOP, namely that the former is the only one that has been mandated—or at least strongly recommended—in some jurisdictions. Survey results on the state of FHIR show that the FHIR standard has been mandated or advised in 20 countries [9]. Notably, the European Electronic Health Record Exchange Format, introduced by the European Commission in 2019 with the aim to ensure secure, interoperable, cross-border access to electronic health data across the European Union, decided in 2022 to adopt Health Level 7 FHIR as the exchange format for future priority data categories [10]. In the United States, the Office of the National Coordinator for Health Information Technology and the Centers for Medicare and Medicaid Services have introduced a steady stream of new regulations, criteria, and deadlines in Health IT that has resulted in significant adoption of FHIR [11]. In India, the open Health Claims Exchange protocol specification—which is based on FHIR—has been mandated by the Indian government as the standard for e-claims handling [12,13]. The African Union recommends all new implementations and digital health system improvements use FHIR as the primary mechanism for data exchange [14], but does not say anything about the use of, for example, openEHR for clinical point-of-service systems.

Our third critical reflection on choosing health data standards pertains to the notion of the hourglass model [4,5] and the concept of open architectures [15]. The hourglass model is “...an approach to design that seeks to support a great diversity of applications (at the top of the hourglass) and allow implementation using a great diversity of supporting services (at the bottom)” [5]. The center of the hourglass—the waist, also called the spanning layer in information systems parlance—is defined by a set of minimal standards that mediates all interactions between the higher and lower layers. In the case of the internet, the spanning layer is defined by the transmission control protocol/internet protocol, which is supported by a variety of underlying connectivity services (many different physical networks) on top of which many different applications can be built (email, videoconferencing, etc). We argue that FHIR has an added benefit over openEHR and OMOP because it can act as the spanning layer within an open health data platform. Because FHIR is inherently designed to function as a data exchange standard, it can function as a mediator between different components of the health data platform. The modularity of the various components that are part of the FHIR ecosystem allows it to be used effectively to implement subsystems, including data pipelines and data processing engines (Textbox 2).

**Conceptual background of the digital platform.**

Digital platforms are software-based digital infrastructures that facilitate interactions and transactions between users. In the context of this paper, digital platforms serve as an interface used to interact with data systems. Data systems describe a set of technologies, tools, and processes that extract, manage, and deliver data. Where the data system describes the functional implementation, the data architecture specifies the design framework, outlining how the data flows in its collection, storage, processing, and governance. Its key components are data sources (original “raw” data that is collected before any processing), data repositories like databases, data warehouses, or lakes, and data processing engines and pipelines that transform raw data into a usable format for analysis.

All architectures include a core technical platform (the foundational infrastructure) that can be extended to facilitate the necessary digital services. Data architectures contain different levels of specifications for the technical components entailed in the system. These levels include a systems code base (machine-readable text describing how to extract and process certain data), software tools (programs and applications enabling digital operations), and stacks (layers of software systems working together).

**Conceptual background of data processing pipelines for analytics.**

Data pipelines define a sequence or workflow of processes for data. Data processing engines are tools that process, transform, and analyze large-scale data and thereby provide the foundational infrastructure to implement data pipelines. Computing workloads are specific tasks executed across data systems, like data processing and analytics.

Data transformation entails all the processing pipelines that convert data into usable insights. Mappings are specific data transformations that aim to align data from different sources with a unified structure. Granular mappings transform data at the most detailed level, translating data elements across different schemas. Queries are built on top of transformed data, and retrieve data for insights generation, sometimes requiring further data processing.

We argue that (1) the external incentives that have mandated FHIR in certain jurisdictions and (2) the inherent modularity of the FHIR standard have resulted in a large boost in both commercial and OSS development activities in the FHIR ecosystem. Illustrative of this is the speed with which the Bulk FHIR API has been defined and implemented in almost all major implementations [16,17], and the SQL-on-FHIR specification to make large-scale analysis of FHIR data both accessible to a larger audience, as well as portable between systems [18].

The external incentives have also led to more people voluntarily contributing to FHIR-related OSS projects, which has resulted in a wide offering of FHIR components across major technology stacks (Java, Python, .NET), thereby strengthening the first condition for establishing openness. By comparison, OMOP and openEHR have profited less from external incentives to spur the adoption and thereby grow the ecosystem beyond a certain critical mass. To illustrate this, a quick scan of the available OSS components listed on the website of the three governing bodies, Health Level 7 [19], Observational Health Data Sciences and Informatics [20], and openEHR [21], indicates that the ecosystem of FHIR and OMOP have a significantly larger offering of extensible and complementary OSS components than openEHR, although for the latter, a notable mature OSS implementation is available with EHRbase [22]. Taking GitHub as a proxy of worldwide development activities, Table 1 shows the number of contributors and repositories for three different search terms. Given that the FHIR standard has more application areas, one would expect more GitHub projects than openEHR and hence these numbers should only be taken as rough indicators.

In summary, we stress that beyond evaluating the intrinsic structure of an open standard and the community that supports the standard, we need to consider the wider ecosystem of OSS implementations and the availability of complementary components. From this wider perspective of the ecosystem surrounding the three standards, FHIR stands out as having the most diverse and rich ecosystem because it has been mandated in certain jurisdictions and because its technical foundations are inherently broader and more modular. This is relevant when comparing these standards in real-world implementations. We now turn to two specific use cases where these considerations are at play.

**Table 1:** Number of contributors and number of repositories on GitHub for the three health care data standards as of January 28, 2025.

Period and search term		Contributors, n	Repositories, n
**Last three months**			
	“openEHR”	82	49
	“OMOP” or “OHDSI”	446	221
	“FHIR”	1648	756
**All time**			
	“openEHR”	429	450
	“OMOP” or “OHDSI”	1019	113
	“FHIR”	8497	8617

## Standardization of Health Data for FL

The current fragmentation of health data is one of the major barriers to leveraging the potential medical data for machine learning (ML). Without access to sufficient data, ML will be limited in its application to health improvement efforts, and ultimately, from making the transition from research to clinical practice. High-quality health data, obtained from a research setting or a real-world clinical practice setting, is hard to obtain because health data is tightly regulated.

**Figure 1:**
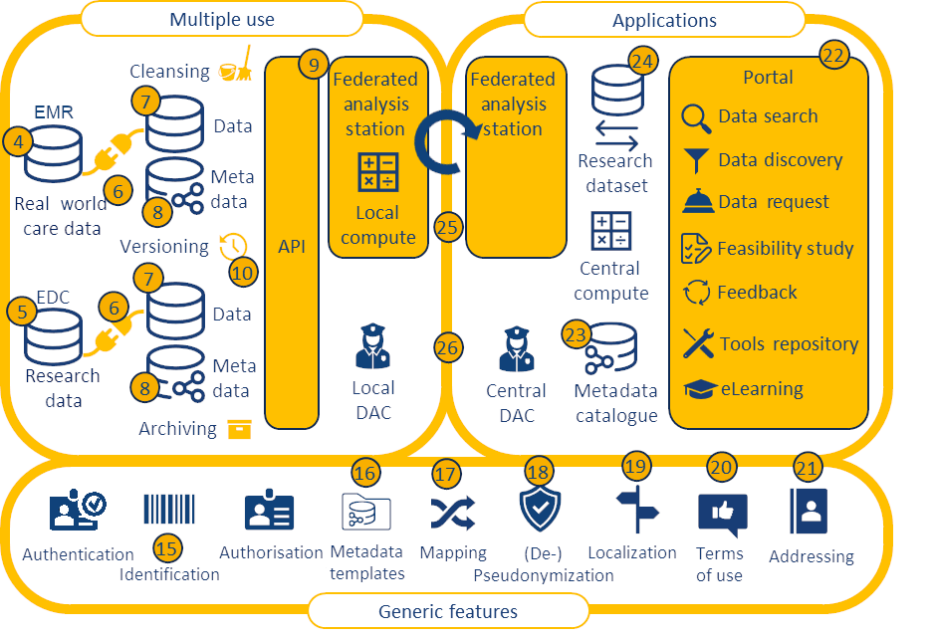
Reference architecture for the Dutch health data infrastructure for research and innovation (reproduced from Health-RI [26], with permission from Health-RI). API: application programming interface; DAC: data access controller; EDC: electronic data capture; EMR: electronic medical record.

FL is a learning paradigm that aims to address these issues of data governance and privacy by training algorithms collaboratively without moving (copying) the data itself (Textbox 3) [23,24]. Based on ongoing work with the PLUGIN health care consortium [25], we have detailed an architecture for FL for the secondary use of health data for hospitals in the Netherlands. The starting point for this implementation is the National Health Data Infrastructure agreements for research, policy, and innovation for the Dutch health care sector, which were adopted at the beginning of 2024 [26]. Figure 1 shows a high-level reference architecture of the infrastructure to be, comprising three areas (multiple use, applications, and generic functions and a total of 26 functional components [26]. One of the prerequisites of this architecture is that organizations that participate in a federation of “data stations” use the same common data model to make the data Findable, Accessible, Interoperable, and Reusable (FAIR). These FAIR data stations comprise components 7, 8, and 9 in Figure 1, that is, the data, metadata, and APIs, respectively, through which the data station can be accessed and used.

Following the line of reasoning of Tsafnat et al [1], OMOP would be the go-to standard for storing the longitudinal data in each of the data stations, where data are transformed from the original source (component 6), stored using a common data model (component 7) and properly annotated with metadata (component 8). Indeed, by now, there are quite a real-world implementations of FL networks based on the Observational Health Data Sciences and Informatics-OMOP stack, including a global infrastructure with 22 centers for COVID-19 prediction models [27], FeederNet in South Korea with 57 participating hospitals [28], Dutch multicohort dementia research with 9 centers [29], the European severe heterogeneous asthma research collaboration [30], and the recently initiated Belgian Federated Health Innovation Network [31].

For the PLUGIN project, however, we choose to adopt FHIR as a data model because it is more compatible with the data model of the clinical administration systems. As PLUGIN focuses on the secondary use of routine health data, we feel it is more suitable than OMOP, the latter being more suitable for clinical research data. OpenEHR might have been an option, too, if more implementations and complementary components had been available. Another reason for choosing FHIR is its practicality and extensibility to be used in a Python-based data science stack, provenance of RESTful APIs out-of-the-box to facilitate easy integration with the container-based vantage6 FL framework, and the support of many health care terminologies and flexibility through its profiling mechanism [32-34]. Increasingly, other projects have reported the use of FHIR for persistent, longitudinal storage for FL. A scoping review on the use of FHIR for clinical research shows that it is increasingly being used for data preparation, cohort selection, and secondary data sharing [35]. The CODA platform, which aims to implement an FL infrastructure in Canada similar to the PLUGIN project, compared OMOP and FHIR and chose the latter as it has been found to support more granular mappings required for analytics [36]. The fair4health project used FHIR as part of a FAIRification workflow to simplify the process of data extraction and preparation for clinical study analyses [37].

Given that OMOP can, conceptually, be viewed as a strict subset of FHIR, hybrid solutions using a combination of OMOP and FHIR have also been reported, such as the German KETOS platform [38], and the preliminary findings from the European GenoMed4All project, which aims to connect clinical and -omics data [39]. A collaboration of 10 university hospitals in Germany has shown that standardized ETL-processing from FHIR into OMOP can achieve 99% conformance [40], which confirms the feasibility of the solution pattern where FHIR acts as an intermediate sharing standard through which data from (legacy) systems are extracted and made available for reuse in a common data model. One could argue that the distinction between FHIR and OMOP becomes less relevant if data can be effectively stored in either standard. We are hopeful that initiatives like OMOP-on-FHIR indeed will foster convergence rather than a collision between these two standards [41].

**Conceptual background of distributed data systems.**

Data systems often have a centralized architecture, where data are collected in a single repository or location. However, data systems can also distribute the storage and processing of data across different nodes or locations such as servers and edge devices.

Servers act as the central processing units in data architecture, supporting computing workloads in data extraction, storage, and transformation of data. Edge devices mainly provide support for data extraction and preprocessing, generally located near the source of the data.

Federated learning is an approach where machine learning (ML) models are trained across a distributed data system. Data transformations and analyses are performed on locally held data across multiple nodes, typically using edge devices or local servers. In this setup, the server that hosts the ML model does not need direct access to the source data. Instead, it aggregates the outputs of the local nodes (the updated model parameters) to train a global model. This method ensures that sensitive data remains local, preserving privacy while still enabling collaborative model training across distributed systems.

**Figure 2:**
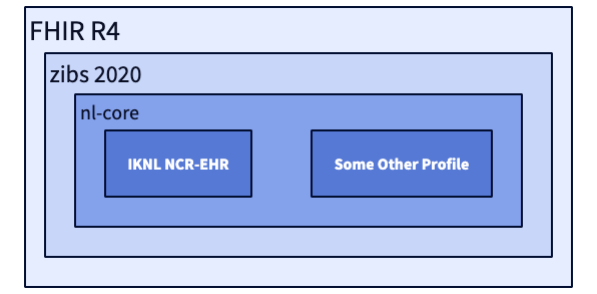
Principle of late binding with FHIR profiling mechanism, illustrated with FHIR profiles that are currently in use in the Netherlands. Each of the successive profiles are concentric, where the two most inner profiles are illustrated to cover different subsets of the parent profile. In practice, the two inner profiles will have an overlap as they, for example, will both include the Patient resource. FHIR: Fast Health Interoperability Resources.

In the case of PLUGIN, another important consideration for choosing FHIR over OMOP is, that from a data architecture perspective, the mechanism of FHIR Profiles can be tied to the principle of late binding commonly applied in data lake or warehouse architectures (Figure 2): allow ingest of widely different sources, and gradually add more constraints and validations as you move closer to a specific use case. If ML is the primary objective for secondary use, one wants to be able to cast a wider net of relevant data, rather than being too restrictive when ingesting the data at the start of the processing pipeline. Late binding in data warehousing is a design philosophy where data transformation and schema enforcement are deferred as late as possible in the data processing pipeline, sometimes even until query time. This approach contrasts with early binding, where data is transformed and structured as it is ingested into the data warehouse. The advantage of this design is that it allows for greater flexibility and allows us to leverage new standards and technologies using the lakehouse architecture and the composable data stack for the implementation of the data stations (Textbox 4). During the initial ingestion of the data, we only require the data to conform to the minimal syntactic standard defined by the base FHIR version (R4 in Figure 2). As the data is processed, more strict checks and constraints are applied, whereby ultimately different profiles can coexist next to one another (the two most inner rectangles in Figure 2), within a larger rectangle with fewer restrictions. Note that if any of the profiles includes a FHIR extension, such as adding a field to include a birth name, the profiles are no longer strictly concentric. Hence extra care needs to be taken when dealing with extensions when applying the principle of late binding.

One of the key challenges in using FHIR in this way pertains to the need for upgrading the whole extract-load-transform pipeline when upgrading to a new primary FHIR version, for example, R6. The potential technical debt of version upgrades in the future is not specific to FHIR, but being a younger standard changes are more frequent compared to OMOP and openEHR. However, we expect that the development time required to upgrade FHIR versions is significantly less than the initial migration to FHIR.

The considerations above also show the conceptual difference between FHIR as a health data exchange standard versus openEHR as a persistent storage of routine health care data and OMOP as a persistent storage of health research data. For health data exchange and FL, the recipient of the data determines, to a large extent, what subset of data in the source needs to be made available, that is, the target data model is known late and this favors late binding. In the case of routine collection of data, the holder of the source data determines what data needs to be stored—and typically everything—which favors early binding.

**Lakehouse architecture and the composable data stack.**

Data lakehouses typically have a zonal architecture that follows the extract-load-transform (ETL) pattern where data is ingested from the source systems in bulk (E), delivered to storage with aligned schemas (L), and transformed into a format ready for analysis and reuse (T) [42-44]. The discerning characteristic of the lakehouse architecture is its foundation on low-cost and directly accessible storage that also provides traditional database management and performance features such as ACID (Atomicity, Consistency, Isolation, and Durability) transactions, data versioning, auditing, indexing, caching, and query optimization [45].

The composable data stack [46] is a new set of technologies and open standards for the fast processing of data using columnar data formats, including Apache Arrow as the standard columnar in-memory format with remote procedure call–based data movement [47]; Apache Parquet as the standard columnar on-disk format [48]; and Apache Iceberg as the open table format [49,50]. This design also enables the use of new embedded, in-memory data processing engines. In turn, this opens up possibilities to bring computing workloads to edge devices, such as running DuckDB in the browser on top of WebAssembly [51].

Using these technologies, full separation of storage and compute can be achieved which allows for cost-effective implementation of data stations.

## Health Data Standards in LMICs

**Figure 3:**
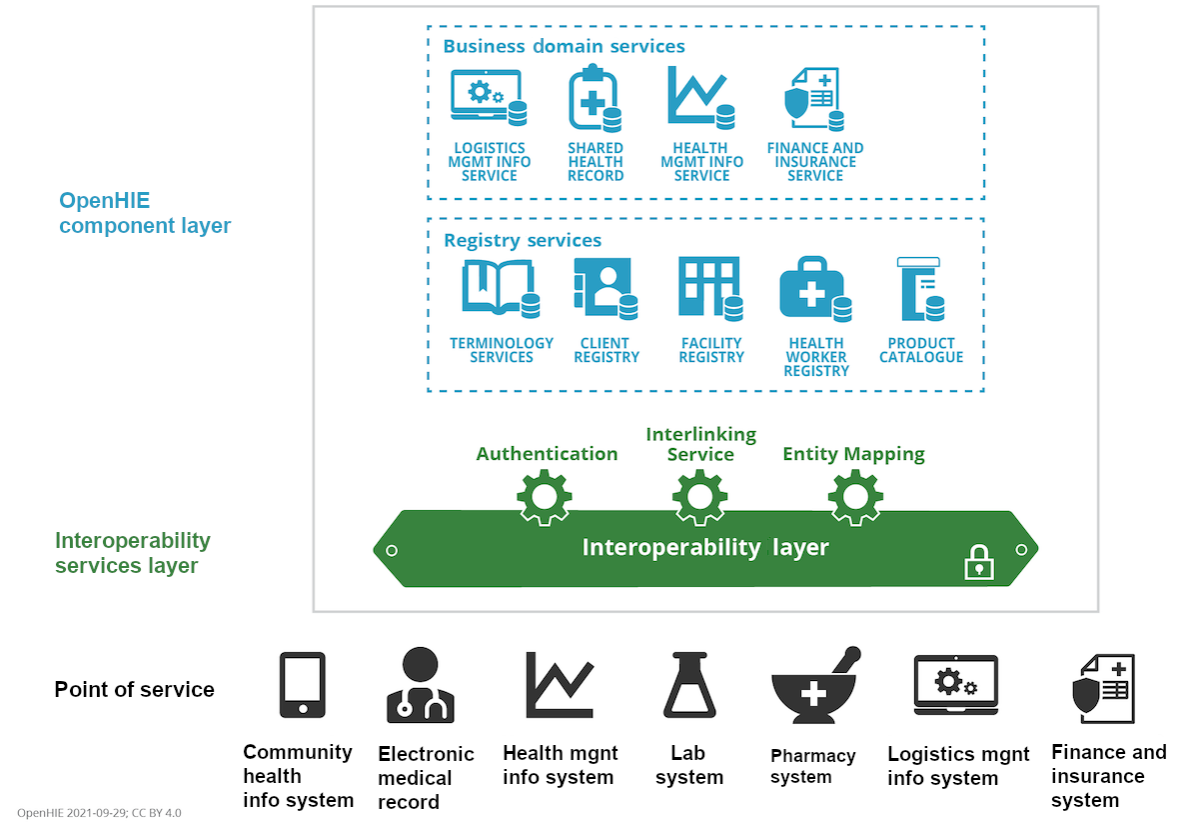
OpenHIE architecture showing the point of service systems (black), the interoperability Layer (green), and the component layer (blue).

It is a widely held belief that digital technologies have an important role to play in strengthening health systems in LMICs. Yet, also here, the current fragmentation of health data stands in the way of scaling up digital health programs beyond project-centric, vertical solutions into sustainable health information exchanges (HIEs) [52]. In the context of global digital health developments, Mehl et al [15] have also called for convergence to open standards, similar to Tsafnat et al [1], but additionally stress the need for OSS (as our main argument in this paper), open content (representations of public health, health system or clinical knowledge to guide implementations), and open architectures (reusable enterprise architecture patterns for health systems). As for the open architecture, we see a convergence toward the OpenHIE framework [53], which has been adopted by many African countries as the architectural blueprint for implementing nationwide HIEs [54], including Nigeria [55], Kenya [56], and Tanzania [57]. Figure 3 shows an overview of the OpenHIE architecture.

**Figure 4:**
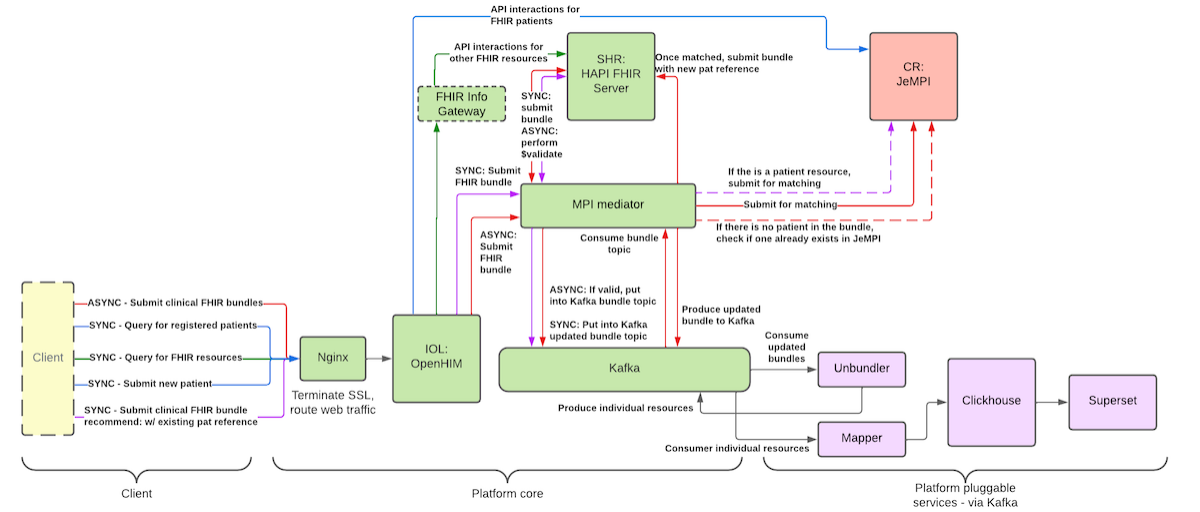
OpenHIM Platform Architecture, illustrating the use of FHIR-based workflows between the components as specified in OpenHIE. API: application programming interface; ASYNC: asynchronous; CR: client registry; FHIR: Fast Health Interoperability Resources; IOL: interoperability layer; MPI: master patient index; SHR: shared health record; SYNC: synchronous.

While the OpenHIE specification is agnostic to which data standards should be used, in practice, the digital health community in LMICs has converged toward FHIR as the primary standard for HIE, in line with the proposal by Tsafnat et al [1]. To illustrate this point, consider the OpenHIM Platform architecture (Figure 4), which is currently the largest OSS implementation of the OpenHIE specification. In OpenHIM, clients (point-of-service systems) can initiate various workflows to submit or query patient data. The shared health record (SHR) acts as the core transactional system for the HIE, which in this case is realized with the HAPI FHIR server, being one of the most widely used open-source FHIR server implementations [58].

**Figure 5:**
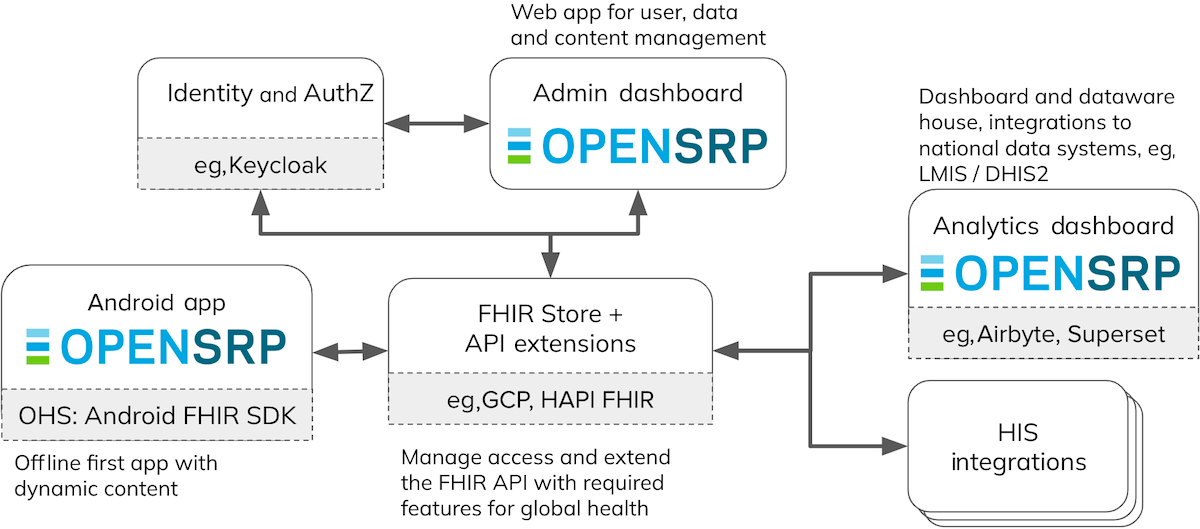
Overview of OpenSRP2 OSS framework for building clinical administration apps. API: application programming interface; FHIR: Fast Health Interoperability Resources; HIS: health information systems.

Looking at the point-of-service systems, we see that as of today, openEHR is rarely used as the standard for routine collection of clinical data in LMICs. The largest OSS electronic health record (EHR) implementations for low-resource settings are based on nonstandardized data models, and it is unlikely this will change any time soon [59]. Instead, we see that FHIR-native software development frameworks such as OpenSRP [60] and the Open Health Stack [61] are being used more and more. In this approach, health professionals use Android apps to register and collect routine health data (Figure 5). As an example, OpenSRP has been deployed in 14 countries targeting various patient populations, among which is the implementation of the WHO antenatal and neonatal care guidelines for midwives in Lombok, Indonesia [62,63]. Beda EMR takes a similar approach and provides an FHIR native front-end that can be used in combination with any FHIR server as a backend [64]. Such a solution design is particularly useful for midsize and smaller health care facilities, which are often resource-constrained, and lacking the basic IT infrastructure to deploy a full-blown electronic medical record system. Hence, the FHIR-based SHR functions as both, the administrative system-of-record and as the hub for information exchange at the same time.

Finally, regarding longitudinal data analysis, we also see a convergence toward FHIR as the primary standard in LMICs. As in the case of FL, the choice for FHIR to implement data warehouse and analytic platforms is the preferred method due to the widespread availability of complementary OSS components. FHIR-specific technologies such as Bulk FHIR data access and SQL-on-FHIR mentioned earlier, allow the FHIR ecosystem to be used, complemented, and integrated with generic OSS data warehousing components such as Clickhouse [65] and dbt [66]. Recently, more studies have pointed to the potential that FHIR brings when it is used in conjunction with ML and artificial intelligence [67]. FHIR-based SHRs can act as systems of records for countries, thereby enabling reuse by health researchers, foundations, etc, to create public value with this data.

All in all, we see that in the context of LMICs, the standardization of the three domains put forward by Tsafnat et al [1] merge into one. The SHR, as the key component within the OpenHIE specification, serves as the back-end of the system of record and provides a transactional, persistent storage engine for information exchange. Downstream longitudinal data stores continue to use FHIR as the common data model for analytical purposes. One could argue that it is in fact advantageous to converge to just one standard, thereby reducing the complexity and cost of the total system. Such a perspective ties in with the notion of the hourglass model and open architectures: because FHIR is inherently designed to make optimal use of internet standards, such as the JSON file format and REST APIs, it is very modular and developer-friendly. The many components that make up the FHIR allow the standard to be used effectively to implement subsystems, such as a facility registry or a health worker registry. By comparison, OMOP and openEHR are designed with a smaller scope with fewer application areas and are thereby less suitable as a standard to implement the subsystems defined in the OpenHIE specification.

## Discussion, Conclusion, and Future Research

We agree with Tsafnat et al [1] that there is a dire need to converge to open data standards in health care and support their proposal to focus on openEHR, FHIR, and OMOP in health care informatics going forward. However, open standards are a necessary but not sufficient condition for the convergence of health data standardization. The availability of OSS implementations and complementary technologies is important when choosing which open standard to use. We find that the proposed trichotomy is too restrictive and therefore of limited use in guiding design choices to be made in real-world scenarios. Instead, we think that the full-STAC approach described by Mehl et al [15] is more comprehensive. Furthermore, we argue that FHIR has the potential to act as the spanning layer for health data interoperability, thereby enabling much wider standardization and adoption within the health data ecosystem at large. This is illustrated by the two cases considered in this paper, where FHIR is used beyond its original scope as a health data exchange standard.

In the case of FL, FHIR can be used interchangeably with OMOP for longitudinal analysis. In addition, due to its inherently modular design, FHIR can be used in conjunction with the principle of late binding, as opposed to early binding for OMOP and openEHR, which is a relevant design criterion for implementing federated data platforms for secondary use. In the case of LMICs, we see that FHIR is emerging as the standard for all three domains of routine health data collection at the clinical point-of-service, data exchange, and longitudinal analysis. We believe this is driven by the resource-constrained setting in LMICs, the modularity of FHIR, and the lower complexity and shallower learning curve of FHIR compared to openEHR. We expect that FHIR will play a major role in driving health data convergence in LMICs because the availability of OSS implementations and complementary components are important enablers in these resource-constrained environments. We strongly support ongoing developments to increase the availability of OSS implementations as digital public goods [68] and integration projects such as Instant OpenHIE [69], which will improve health data interoperability in LMICs.

Although openEHR has not been chosen as the standard for the two use cases presented here, we want to stress that it is not our intention to argue for or against any of the three standards a priori nor do we intend to dismiss openEHR outright. Instead, our aim is to illustrate the kind of design choices and trade-offs that need to be made, particularly those related to the availability and complementarity of OSS components. Significant developments and uptake of openEHR as a clinical data repository have been reported, with currently 17 openEHR solution providers that have been implemented in thousands of clinics and research organizations worldwide [70]. Additionally, work is underway to integrate openEHR with FHIR for data exchange [71,72]. Some experts agree that openEHR is the only specification that provides a comprehensive solution for building a standardized EHR [73]. Furthermore, openEHR is not only being deployed as a clinical system of record but also as a persistent clinical data repository for implementing national HIEs in European Nordic countries [74] and Slovenia [75], which is very similar to the solution design of the SHR within the OpenHIE architecture presented here. An ongoing program in the south of the Netherlands has demonstrated a decentralized data sharing ecosystem using separate openEHR data stores, where federated queries are supported by the openEHR Archetype Query Language [76].

The two cases allow us to reflect and revisit the key arguments of this paper, namely the importance of OSS implementations and the availability of complementary components for the wide-scale adoption of health data standards. There is an important and equally complex interplay between OSS development and standardization, where OSS implementation can occur before, after, or in parallel to standardization efforts [77,78]. Various studies have provided increasing evidence that OSS is a key success factor in driving software-related standardization [78], and by extension, we think it is critical when aiming to achieve data standardization. The history of how the Digital Imaging and Communications in Medicine imaging standard came to be is a good example of how OSS development was pivotal in achieving wide-scale adoption of this standard [79,80].

In contrast, the phenomena of forking, fragmentation, and splintering are known to hinder an industry from consolidating toward a set of open standards [81]. Given the specific characteristics of data as an artifact, fragmentation is arguably the most relevant of these phenomena. de Reuver et al [6] expect fragmentation to persist for some time in the evolution of data platforms and associated ecosystems. The case of the Unix operating system is an interesting example where fragmentation hampered standardization, next to market dynamics and issues related to intellectual property rights [81].

But even when OSS has successfully contributed to “tip” the health care industry to a set of health data standards, issues remain regarding the sustainability of the OSS ecosystem itself. The market dynamics and economics of OSS ecosystems differ considerably from industry to industry: sustainability of OSS in the context of, say, the cultural and scientific heritage sector will be different from the challenges of OSS projects that are used as mission-critical components of open digital infrastructures worldwide. In the case of the latter, underfunding is a critical issue and initiatives such as the German Sovereign Tech Agency have been launched to alleviate this [82]. In the context of open health data standards, we believe that risks related to underfunding are lower and more manageable. Within the digital health community, there is a range of commercial companies supporting the OSS projects and creating sustainable businesses from it using various business models like offering support contracts, split licensing, and complementary closed-source products [83]. Regarding the dynamics of forking and fragmentation mentioned earlier, we feel that code forking on balance has a net positive effect on the long-term sustainability of OSS at the level of the software itself, the community, and the ecosystem [84].

Going forward, we suggest the following directions for future research. Given that health data standardization will continue to require mappings, we propose to explore the use of ML, particularly large-language models, as a means to reduce the development effort required to create transformations between various health data formats. New ML methods can also be developed to assess and improve data quality across the various stages of the data processing pipelines. In terms of data integration, we expect that health data will increasingly be used in conjunction with data from social services and the welfare domain, which requires new techniques to integrate different data domains, for example, using knowledge graphs and ontologies. Last, but certainly not least, future research should not only explore the technical but also the social implications of implementing OSS components for data standardization across the health care system, specifically in settings where governance or ethical considerations of data interoperability have not specifically been addressed at a regulatory level. In line with the embedding of open standards in the open-source ecosystem, we assert that the benefits of health data standardization will only be realized if they are coupled with collaborative, community-driven governance models. It remains essential to ensure that the development, adoption, and evolution of standards remain inclusive, transparent, and responsive to the diverse needs within the health system.

## Abbreviations

EHR: electronic health record
FAIR: Findable, Accessible, Interoperable, and Reusable
FHIR: Fast Health Interoperability Resources
FL: federated learning
HIE: health information exchange
LMIC: low and middle-income country
ML: machine learning
OMOP: Observational Medical Outcomes Partnership
OSS: open-source software
SHR: shared health record
